# Venous Thromboembolism Risk Assessment and Prophylaxis in Obstetrics Patients in a Tertiary Health Center in Jeddah, Saudi Arabia

**DOI:** 10.7759/cureus.63741

**Published:** 2024-07-03

**Authors:** Ahmed M Noorelahi

**Affiliations:** 1 Internal Medicine, King Abdulaziz University Hospital, Jeddah, SAU

**Keywords:** obesity, pregnancy, thromboprophylaxis, risk assessment, venous thromboembolism

## Abstract

Aim

The study aimed to estimate the proportion of patients who were evaluated for thrombosis risk and received appropriate thromboprophylaxis, if indicated, in King Abdulaziz University Hospital (KAUH), Jeddah, Saudi Arabia.

Method

This was a retrospective cross-sectional study conducted among inpatients from May 1 to August 31, 2020, in KAUH.

Results

Out of 298 pregnant women, the mean age was 32.09 ± 5.29 years. A total of 136 (45.6%) were obese and 97 (32.6%) were overweight. There was a significant relationship between Caprini score categories and the following variables: age, body mass index (BMI), medical disease, history of deep vein thrombosis (DVT), mode of delivery, prophylaxis, and its duration (p < 0.05).

Conclusion

Awareness about thromboprophylaxis in reducing the risk of developing venous thrombosis has increased compared to before, with more than half of the cases receiving it regardless of the risk level. The highest risk of venous thromboembolism (VTE) was found in older women, obese women, and those with a previous history of DVT. Low-molecular-weight heparin (LMWH) was the main prophylaxis in the hospital with an average duration of three days.

## Introduction

Obstetric-associated venous thromboembolism (VTE) is an important, yet preventable, cause of mortality and morbidity during pregnancy and the postpartum period [1,2].

Pulmonary embolism (PE) is considered the second leading cause of maternal death in Saudi Arabia [3]. In addition, the risk of deep vein thrombosis (DVT) increases five to 10 times during pregnancy, and this elevated risk may persist for up to 12 weeks postpartum [2]. In developed countries, the incidence of VTE during pregnancy ranges between one and two cases per 1,000 pregnancies [4]. In Saudi Arabia, the reported incidence of VTE during pregnancy and the puerperium is 1.25 cases per 1,000 deliveries [5]. Moreover, the incidence of VTE is found to be higher in women during their childbearing years [6]. VTE is a multifactorial disease that manifests clinically as DVT and PE [7]. Risk factors for VTE in obstetric patients include multiparity, obesity, advanced maternal age, repeated cesarean sections, and consanguineous marriages, with the majority of the obstetrical population in Saudi Arabia being marked as high-risk [5]. In addition, hospitalized women have various risk factors for developing VTE [8]. Therefore, VTE risk assessment and thromboprophylaxis for high-risk groups can dramatically minimize PE-related mortality [9]. Despite decades of attention and well-publicized guidelines, the utilization rates of VTE risk assessment and prophylaxis remain suboptimal globally [10-12].

In Saudi Arabia, thromboprophylaxis is underutilized in 50% of obstetric patients [5]. Therefore, this study aims to estimate the proportion of patients at King Abdulaziz University Hospital (KAUH) in Jeddah, Saudi Arabia, who were assessed for thrombosis risk and received appropriate thromboprophylaxis when indicated.

## Materials and methods

This retrospective cross-sectional study was conducted among inpatients at KAUH from May 1, 2020, to August 31, 2020. KAUH, a teaching tertiary center located in Jeddah, Saudi Arabia, is renowned for its comprehensive healthcare services and important educational contributions.

Ethical approval for this study was obtained from the Institutional Review Board (IRB) of KAUH (Reference No. 250-15). The study adhered to the ethical standards outlined in the Declaration of Helsinki, ensuring the protection of patient rights and maintaining data confidentiality.

Data were retrospectively collected from the hospital's electronic medical records system. The study included obstetric inpatients with a length of stay exceeding one day, who were evaluated for thrombosis risk during the specified period from May 1, 2020, to August 31, 2020. Patients already receiving anticoagulant therapy upon admission and those admitted with a primary diagnosis of VTE were excluded. The electronic medical records offered extensive patient data, ensuring the precision and dependability of the collected information.

Thrombosis risk was evaluated using the Caprini risk assessment model, a validated tool employed in clinical practice since 2005 [13]. The Caprini score is calculated based on various risk factors, such as patient age, surgical history, presence of malignancy, and other comorbidities, such as stroke, acute myocardial infarction, prior episodes of VTE, positive family history of VTE, sepsis, varicose veins, hormonal therapy, oral contraceptive, and bed rest. Patients were classified into four risk categories based on their Caprini scores: low risk (0-1), moderate risk (2), high risk (3-4), and highest risk (≥5). This stratification enabled the classification of patients according to their likelihood of experiencing thrombotic events.

Data were systematically recorded using structured Google Forms (Google LLC, Mountain View, California, United States), which encompassed various fields: patient information (file number and demographics, such as age, gender, weight, height, and BMI), clinical details (admission diagnosis, active diseases, and past medical history including previous VTE), thrombosis risk assessment (Caprini score and its components), and details regarding prophylaxis (type of prophylaxis administered, such as low-molecular-weight heparin (LMWH), unfractionated heparin (UFH), warfarin, aspirin, and mechanical devices, including the dose, route of administration, and duration). This structured approach ensured comprehensive and consistent data collection across all patients included in the study.

Data analysis was conducted using the IBM SPSS Statistics, Version 21.0 (released 2012, IBM Corp., Armonk, NY). Descriptive statistics were utilized to summarize quantitative data, presenting frequencies (numbers and percentages) for categorical variables. A crosstab analysis was performed to investigate the relationship between patients' thrombosis risk levels and the administration of VTE prophylaxis. This analysis aimed to identify patterns and evaluate the appropriateness of prophylactic measures based on risk stratification. The chi-square test was employed to assess the statistical significance of the relationship between categorical variables. A significance level of p < 0.05 was considered statistically significant, indicating a meaningful relationship between the variables under investigation.

## Results

As depicted in Table 1, among 298 pregnant women, 266 (89.3%) were Saudi nationals, with a mean age of 32.09 ± 5.29 years. Less than half, 136 (45.6%), were classified as obese (BMI ≥30) across various classes, and 97 (32.6%) were overweight (BMI 25-29.9). Patients with a BMI of 18.5-24.9 were considered normal weight. Table 1 summarizes the demographic characteristics of the patients.

**Table 1:** Demographic characteristics of the patients (n= 298) Data were expressed as frequency (%) or mean ± standard deviation (minimum-maximum).

Demographic characteristics	N (%)
Nationality:	
Saudi	266 (89.3%)
Non-Saudi	32 (10.7%)
Body mass index (BMI) category:	
Underweight	5 (1.7%)
Normal weight	60 (20.1%)
Overweight	97 (32.6%)
Class 1 obesity	77 (25.8%)
Class 1I obesity	34 (11.4%)
Class 1II obesity	25 (8.4%)

As shown in Table 2, less than a quarter (20.5%) of the patients had a chronic illness. Only three cases had a previous history of DVT. More than half (60.1%) underwent a cesarean section. Regarding primary diagnoses on admission and Caprini score categories, LMWH was administered as prophylaxis in 180 (60.4%) cases, with a common dose of 40 mg given in 168 (56.4%) instances, and an average duration of 3.39±5.89 days, as presented in Table 3.

**Table 2:** Obstetric characteristics of the patients (n = 298)

Characteristics	N (%)
Comorbidity	
No	237 (79.5%)
Yes	61 (20.5%)
Admission diagnoses:	
Supervision of normal pregnancy	159 (53.3%)
Supervision of high-risk pregnancy	24 (8.1%)
Maternal care due to uterine scar from other previous surgery	63 (21.1%)
Maternal care for breech presentation	13 (4.4%)
Multiple gestations	14 (4.7%)
Premature rupture of membrane	6 (2.0%)
Placenta previa with hemorrhage	5 (1.7%)
Diabetes mellitus arising during pregnancy	5 (1.7%)
Pre-existing diabetes mellitus in pregnancy	3 (1.0%)
Others:	6 (2.0%)
History of deep venous thrombosis	
No history	295 (99.0%)
History of pulmonary embolism	1 (0.3%)
History of deep venous thrombosis and pulmonary embolism	2 (0.7%)
Caprini score	
Low risk	141 (47.3%)
Moderate risk	113 (37.9%)
High risk	39 (13.1%)
Highest risk	5 (1.7%)
Mode of delivery	
Spontaneous vaginal delivery (SVD)	119 (39.9%)
Cesarean section (CS)	179 (60.1%)

**Table 3:** Prophylaxis The data were expressed as frequency (%) or mean ± standard deviation (minimum-maximum).

Characteristic	N (%)
Prophylaxis:	
No	118 (39.6%)
Yes, Clexane	180 (60.4%)
Clexane dose:	
40 mg	168 (56.4%)
60 mg	9 (3.0%)
80 mg	3 (1.0%)

The admission diagnoses were categorized into different categories, as presented in Table 4 and Table 5.

**Table 4:** Supervision of pregnancy and maternal care

Supervision of pregnancy and maternal care	N (%)
Supervision of normal pregnancy	159 (53.3%)
Supervision other normal pregnancy	101 (33.9%)
Supervision of normal pregnancy, unspecified	10 (3.4%)
Pregnant state, incidental	11 (3.7%)
Supervision of normal first pregnancy	8 (2.7%)
Unspecified duration of pregnancy	7 (2.3%)
Prolonged pregnancy	6 (2.0%)
Duration of pregnancy 34-36 completed weeks	5 (1.7%)
Supervision of pregnancies with a history of infertility	2 (0.7%)
Duration of pregnancy 20-25 weeks completed	1 (0.3%)
Supervision of normal pregnancy	4 (1.3%)
Pregnancy confirmed	2 (0.7%)
Supervision of primigravida with advanced maternal age	1 (0.3%)
Maternal care for high head at term	1 (0.3%)
Maternal care due to uterine scar from other previous surgery	63(21.1%)
Maternal care due to uterine scar from other previous surgery	59 (19.8%)
Single delivery by caesarean section	4 (1.3%)
Supervision of high risk pregnancy	24(8.1%)
Supervision of other high risk pregnancy	13 (4.4%)
Supervision of high risk pregnancy, unspecified	11 (3.7%)
Maternal care for breech presentation	13(4.4.%)
Maternal care for breech presentation	12 (4.0%)
Labor and delivery affected by breech presentation	1 (0.3%)
Multiple gestation	
Triplet pregnancy	3 (1.0%)
Twin pregnancy	11 (3.7%)

**Table 5:** Other maternal conditions and complications The data were expressed as frequency (%) or mean ± standard deviation (minimum-maximum).

Other maternal conditions and complications	N(%)
Premature rupture of membrane	6 (2.0%)
Premature rupture of membrane, unspecified	4 (1.3%)
Premature rupture of membrane, onset of labor within 24 hours	2 (0.7%)
Placenta Previa with hemorrhage	5 (1.7%)
Placenta previa specified as without hemorrhage	4 (1.3%)
Placenta previa with hemorrhage	1 (0.3%)
Diabetes mellitus arising during pregnancy	5 (1.7%)
Diabetes mellitus arising during pregnancy, other	2 (0.7%)
Diabetes mellitus arising during pregnancy, unspecified	2 (0.7%)
Diabetes mellitus arising during pregnancy, insulin treated	1 (0.3%)
Pre-existing diabetes mellitus in pregnancy	3 (1.0%)
Preexisting diabetes mellitus in pregnancy, unspecified, non-insulin treated	1 (0.3%)
Pre-existing diabetes mellitus, type 2, in pregnancy, non-insulin treated	1 (0.3%)
Preexisting diabetes mellitus, type 2, insulin treated	1 (0.3%)
Others	6 (2%)
Hodgkin disease, lymphatic predominance	1 (0.3%)
Small for gestational age	1 (0.3%)
Bicornuate uterus	1 (0.3%)
Maternal care for other fetal problems	1 (0.3%)
Maternal care for poor fetal growth	1 (0.3%)
Maternal care for intrauterine fetal growth	1 (0.3%)

The results in Table 6 and Table 7 present a significant relationship between the Caprini score categories and several variables: age, BMI, medical history, history of DVT, mode of delivery, type of prophylaxis, and duration of prophylaxis. Older women, obese individuals, those with a previous history of DVT, those undergoing cesarean section, and those receiving longer durations of prophylaxis exhibited higher risk rates compared to others. Table 6 and Table 7 present the relationship between the Caprini score and each variable. Table 6 presents categorical variables, while Table 7 presents continuous variables.

**Table 6:** Relation between Caprini score and each variable (categorical variables) * indicates statistical significance at the p < 0.05 level. ** indicates statistical significance at the p < 0.01 level.

Categorical variables	Risk Level				P value
	Low	Moderate	High	Highest	
Nationality					
Saudi	123 (87.2%)	101 (89.4%)	37 (94.9%)	5 (100.0%)	0.150
Non-Saudi	18 (12.8%)	12 (10.6%)	2 (5.1%)	0 (0.0%)	
Body mass index					
Below weight	2 (1.4%)	3 (2.7%)	0 (0.0%)	0 (0.0%)	0.0001**
Normal weight	55 (39.0%)	2 (1.8%)	2 (5.1%)	1 (20.0%)	
Overweight	84 (59.6%)	12 (10.6%)	0 (0.0%)	1 (20.0%)	
Class 1 obesity	0 (0.0%)	68 (60.2%)	8 (20.5%)	1 (20.0%	
Class 2 obesity	0 (0.0%)	28 (24.8%)	4 (10.3%)	2 (40.0%)	
Class 3 obesity	0 (0.0%)	0 (0.0%)	25 (64.1%)	0 (0.0%)	
Medical disease					
Medically free	118 (83.7%)	93 (82.3%)	23 (59.0%)	3 (60.0%)	0.008*
Known case of any chronic disease	23 (16.3%)	20 (17.7%)	16 (41.0%)	2 (40.0%)	
History of deep venous thrombosis					
No history	141 (100.0%)	113 (100.0%)	39 (100.0%)	2 (40.0%)	0.0001**
History of pulmonary embolism	0 (0.0%)	0 (0.0%)	0 (0.0%)	1 (20.0%)	
History of deep venous thrombosis and pulmonary embolism	0 (0.0%)	0 (0.0%)	0 (0.0%)	2 (40.0%)	
Mode of delivery					
Spontaneous vaginal delivery (SVD)	66 (46.8%)	43 (38.1%)	9 (23.1%)	1 (20.0%)	0.020*
Cesarean section (CS)	75 (53.2%)	70 (61.9%)	30 (76.9%)	4 (80.0%)	
Prophylaxis					
No	67 (47.5%)	43 (38.1%)	8 (20.5%)	0 (0.0%)	0.001*
Clexane	74 (52.5%)	70 (61.9%)	31 (79.5%)	5 (100.0%)	

**Table 7:** Relation between the Caprini score and each variable (continuous variables) * indicates statistical significance at the p < 0.05 level. ** indicates statistical significance at the p < 0.01 level.

Continuous variables	Mean	SD	P-value
Age			
Low	31.1	4.7	0.0001**
Moderate	31.9	5.3	
High	35.8	5.9	
Highest	36.2	3.6	
Duration of prophylaxis (days)			
Low	2.4	0.5	0.0001**
Moderate	2.6	0.8	
High	2.8	0.4	
Highest	26.4	21.0	

## Discussion

VTE remains a primary cause of maternal mortality, despite being a preventable condition. A systematic review of maternal mortality conducted by the World Health Organization highlighted that embolism accounted for 14.9% of maternal deaths in developed countries [14].

This study identified a significant incidence of VTE in 52.7% of patients when comparing those in high- and moderate-risk categories. The rate of thromboprophylaxis administration was 60.8%, which contrasts with findings from a Ghana study where the DVT risk rate was 36.4%, and only 6.1% (5/82) of at-risk participants received VTE prophylaxis [15]. Similarly, Revell et al. reported that a small proportion of at-risk obstetric patients met prophylaxis criteria (7.0% antenatal and 41.0% postnatal patients) [16]. In addition, Alsayegh et al. noted that despite a 32% higher risk of VTE, only 8.3% received recommended prophylaxis [1]. These disparities may stem from various socioeconomic factors, geographic differences, sample sizes, and study methodologies. The low prophylaxis rate could be attributed to insufficient awareness of the important risks and benefits associated with VTE prophylaxis in these cases.

In this study, obesity emerged as an important risk factor associated with the highest risk of DVT (p < 0.0001). This finding aligns with the Arab Gulf study, where obesity rates among pregnant women, excluding Oman, were notably high and approaching epidemic levels [17,18]. Obesity commonly coexists with other conditions necessitating appropriate prophylaxis during pregnancy, thereby compounding the risk of VTE in these cases. While obesity alone has been identified as a risk factor for VTE during pregnancy [19], it does not independently indicate the necessity for VTE prophylaxis unless accompanied by other risk factors. Unfortunately, there is a lack of conclusive studies specifically evaluating the benefits of routine prophylaxis for morbidly obese pregnant women.

In this study, a significant relationship was observed between age and the risk of DVT, with older women exhibiting a higher risk compared to younger women. This finding is consistent with another study that reported an average age of 33.2 years among DVT cases, suggesting the impact of age-related variables on thromboembolic risk [20]. In addition, the Royal College has reported that pregnant women aged 35 years and older are at increased risk of thromboembolic events [20].

In this study, only eight cases had diabetes, including type 2 diabetes or gestational diabetes mellitus. This contrasts with findings from studies in Ghana and Norway, which identified a significant relationship between diabetes mellitus and the risk of DVT [15,21]. Another notable risk factor identified was a previous history of DVT (p < 0.0001), consistent with findings from studies in the Arabian Gulf [17].

This study has several limitations. The retrospective design and single-center setting limit its generalizability to broader populations. The short duration of the study period may not fully capture long-term trends in thromboembolic events. In addition, the sample size might be inadequate to detect all potential risk factors comprehensively. Future research should focus on conducting larger, multicenter studies to address these limitations and provide more robust insights into thrombosis risk and prophylaxis strategies in obstetric patients.

## Conclusions

The study highlights the importance of raising awareness about the benefits of thromboprophylaxis in reducing VTE. It reveals that older age, obesity, and a history of previous DVT are important risk factors for VTE. LMWH was the predominant prophylactic used, typically administered for an average of three days. This underscores the need for targeted preventive measures tailored to high-risk groups. In addition, patient education on recognizing early symptoms of venous thrombosis is crucial for timely intervention. Future nationwide studies should encompass larger sample sizes and diverse hospital settings beyond Jeddah to validate these findings, potentially identifying new risk factors and refining preventive strategies for managing deep venous thrombosis risk effectively.
